# Proteomic analysis of seminal fluid from men exhibiting oxidative stress

**DOI:** 10.1186/1477-7827-11-85

**Published:** 2013-09-03

**Authors:** Rakesh Sharma, Ashok Agarwal, Gayatri Mohanty, Stefan S Du Plessis, Banu Gopalan, Belinda Willard, Satya P Yadav, Edmund Sabanegh

**Affiliations:** 1 Center for Reproductive Medicine, Glickman Urological and Kidney Institute, Cleveland Clinic, Cleveland, OH, USA; 2 Medical Physiology, Stellenbosch University, Tygerberg, South Africa; 3 Bioinformatics Core Services, Lerner Research Institute, Cleveland Clinic, Cleveland, OH, USA; 4 Proteomics Core Services, Lerner Research Institute, Cleveland Clinic, Cleveland, OH, USA; 5 Molecular Biotechnology Core lab, Lerner Research Institute, Cleveland Clinic, Cleveland, OH, USA; 6 Permanent address: Ravenshaw University, Cuttack, Odisha, India

**Keywords:** Seminal plasma, Male infertility, Varicocele, Reactive oxygen species, Oxidative stress, Proteomics, Mass spectroscopy, Differentially expressed proteins, Gene ontology

## Abstract

**Background:**

Seminal plasma serves as a natural reservoir of antioxidants. It helps to remove excessive formation of reactive oxygen species (ROS) and consequently, reduce oxidative stress. Proteomic profiling of seminal plasma proteins is important to understand the molecular mechanisms underlying oxidative stress and sperm dysfunction in infertile men.

**Methods:**

This prospective study consisted of 52 subjects: 32 infertile men and 20 healthy donors. Once semen and oxidative stress parameters were assessed (ROS, antioxidant concentration and DNA damage), the subjects were categorized into ROS positive (ROS+) or ROS negative (ROS-). Seminal plasma from each group was pooled and subjected to proteomics analysis. In-solution digestion and protein identification with liquid chromatography tandem mass spectrometry (LC-MS/MS), followed by bioinformatics analyses was used to identify and characterize potential biomarker proteins.

**Results:**

A total of 14 proteins were identified in this analysis with 7 of these common and unique proteins were identified in both the ROS+ and ROS- groups through MASCOT and SEQUEST analyses, respectively. Prolactin-induced protein was found to be more abundantly present in men with increased levels of ROS. Gene ontology annotations showed extracellular distribution of proteins with a major role in antioxidative activity and regulatory processes.

**Conclusions:**

We have identified proteins that help protect against oxidative stress and are uniquely present in the seminal plasma of the ROS- men. Men exhibiting high levels of ROS in their seminal ejaculate are likely to exhibit proteins that are either downregulated or oxidatively modified, and these could potentially contribute to male infertility.

## Background

The seminal plasma is composed of secretions from accessory sex glands and is rich in sugars, lipids, proteins, and other metabolites that interact with spermatozoa [1,2]. Many seminal plasma proteins can attach to the surface of human spermatozoa and promote motility and prevent premature acrosome reactions [3]. Similarly, heparin binding proteins bind to the sperm surface during ejaculation and form a protein coating [4]. These findings suggest that seminal plasma proteins play a role in fertilization including capacitation, acrosome reaction and oocyte interaction [5].

Advancements in mass spectrometry have aided in the identification of seminal plasma proteins that regulate sperm function. Improvements in proteomic approaches including pre-fractionation of proteins, improved resolution and high sensitivity of mass spectrometry techniques have improved our understanding of the proteomic composition of the seminal plasma [6]. Several techniques have been used to identify the proteins of functional relevance on a comparative basis. This includes the differential in gel electrophoresis approach, which separates intact proteins for quantitative analysis and identification by mass spectrometry analysis. This method has been used to compare the post-translational changes as well as changes in the external sperm coat; synthesis and secretion of some novel glycoproteins resulting in glycosylation and modification of the surface charge etc. are taking place as spermatozoa pass through the epididymal region [7]. The top-down proteomics approach identifies large number of peptides present in the sample; it is advantageous as peptides show a lesser degree of variability than proteins and thus form a reliable basis for proteomic comparisons [8]. Many of the proteomic analysis of bodily fluid such as the seminal plasma have been done using liquid chromatography tandem mass spectrometry (LC-MS/MS) measurements [9]. Although this is a useful method, the accuracy of mass measurement is low and the possibility of identifying unambiguous proteins is high.

The recently developed linear ion trap (LTQ) coupled to a Fourier transform ion cyclotron resonance analyzer has been useful for its high sensitivity and fast sequencing cycles with high mass accuracy and resolution [10]. Several reports have identified the proteins of the seminal plasma, and a repository has been developed. Fung et al. have identified 100 proteins in the seminal plasma of normal individuals utilizing 1-dimensional and 2-dimensional electrophoresis methods coupled to both matrix assisted laser desorption ionization-time of flight (MALDI-TOF) mass spectrometry and liquid chromatography-tandem mass spectrometry (LC-MS/MS) [11]. Similarly, Milardi et al. identified about 1487 proteins in five fertile men using high resolution LTQ-Orbitrap XL hybrid mass spectrometer [5].

Several comparative studies utilizing proteomic approaches have suggested that there are proteins in asthenozoospermia patients that are not present in healthy men [12] or in post varicocelectomy patients [13]. However, information on the unique proteins present in the seminal plasma containing high levels of reactive oxygen species (ROS) is lacking. Oxidative stress has been implicated in the etiology of male infertility [14-21]. Identifying and understanding the proteins that play a key role in oxidative stress-induced pathophysiology of male infertility is important as it can help in the development of some of these key proteins as potential biomarkers.

In this study, we identified the differentially expressed proteins in the seminal plasma of 1) infertile men who were referred to our andrology clinical laboratory for measurement of oxidative stress parameters such as levels of ROS in spermatozoa, concentration of antioxidant in the seminal plasma and the extent of DNA damage 2) healthy men of unproven fertility exhibiting high levels of ROS in their seminal ejaculates and 3) healthy men of unproven fertility with low levels of ROS. We employed LC-MS/MS techniques, and Bioinformatics analysis was performed to understand the roles of the differentially expressed proteins in the seminal plasma of ROS+ and ROS- subjects and their roles in various signaling pathways. Together, the findings from this study should provide a platform to better understand the underlying pathology of oxidative stress in infertile men.

## Methods

Following approval from the Institutional Review Board (IRB) of Cleveland Clinic, we enrolled a total of 52 subjects: 20 healthy male volunteers of unproven fertility (men with normal semen analysis results but who had not yet established a pregnancy) and 32 infertile men attending our infertility clinic who were referred to the andrology lab for evaluation of oxidative stress parameters. IRB consent approved by the Cleveland Clinic was provided to each subject, and the purpose of the study was clearly explained. If the participant was interested, a written signature was obtained and witnessed before he was enrolled in the study.

Of the 32 patients, 25 (25 of 32; 78.1%) presented with primary infertility and 7 (7 of 25; 28%) with secondary infertility. 64% (16 of 25) had primary infertility of ≤2 years and 36% (9 of 25) >2 years. Clinical varicocele (grade 1–2) was seen in 62.5% of patients with primary infertility <2 years and in 55.6% with >2 years. In patients with secondary infertility, 85.7% of the patients were identified with clinical varicocele (grade 1–2). Of the 32 patients, only 10 subjects (10 of 32; 31.3%) had a history of smoking.

Semen samples were collected by masturbation after 2–3 days of sexual abstinence and analyzed according to WHO 2010 criteria [22]. Semen analysis included both macroscopic (volume, pH, color, viscosity, liquefaction time, split or complete ejaculate) and microscopic parameters (concentration, motility, morphology, round cells and peroxidase or Endtz test). All infertile men were also examined for oxidative stress parameters such as levels of ROS in the seminal ejaculate, total antioxidant concentration in the seminal plasma and extent of DNA damage in the spermatozoa. Specimens that were positive for the Endtz test (>1×10^6^ white blood cells/ mL) indicative of an underlying infection were not included in the study. Following these tests, the remaining seminal ejaculate was frozen without any cryoprotectant and stored at −55°C for proteomic analysis.

### Semen analysis

After liquefaction, complete semen analysis was performed to evaluate the sperm parameters (sperm count, percentages motility and morphology), according to WHO guidelines [22]. Semen analysis was done using a MicroCell counting chamber (Vitrolife, San Diego, CA). Smears of the raw semen were stained with a Diff-Quik kit (Baxter Healthcare Corporation, Inc., McGaw Park, IL) for assessment of sperm morphology according to WHO criteria.

### Measurement of reactive oxygen species

ROS levels were measured in seminal ejaculates by chemiluminescence assay using luminol (5-amino-2, 3- dihydro-1, 4-phthalazinedione; Sigma, St Louis, MO). Test samples consisted of luminol (10 μL, 5 mM) and 400 μL of semen. Negative controls were prepared by replacing the sperm suspension with 400 μL phosphate buffered saline. Positive control included 400 μL of PBS and 50 μL of hydrogen peroxide (30%; 8.8 M) in triplicate. Chemiluminescence was measured for 15 min using a Berthold luminometer (Autolumat Plus 953). Results were expressed as relative light units (RLU)/sec/ × 10^6^ sperm [23,24].

### Measurement of total antioxidant capacity

Total antioxidant capacity (TAC) was measured in the seminal plasma samples using an antioxidant assay kit (Cayman Chemical Company, Ann Arbor, MI) as described in our earlier work [25]. Its principle is based on the ability of aqueous- and lipid-based antioxidants in seminal plasma to inhibit oxidation of the ABTS (2,2’-Azino-di-[3-ethylbenzthiazoline sulphonate]) to ABTS^•+^. For the assay, both standard (trolox) and sample (10 μL; 1:10 vol/ vol.) was run in duplicate. 10 μL of metmyoglobin and 150 μL of chromogen were added to each well. The reaction was initiated by addition of 40 μL of hydrogen peroxide (working solution) using a multi-channel pipette as described earlier [25]. The antioxidants present in the seminal plasma suppress absorbance to a degree that is proportional to their concentration. The measurement was read at 750 nm using Microplate Reader (Epoch BioTek Gen 5 Absorbance; BioTek Instruments, Inc., Winooski, VT). The results were expressed as micromole trolox equivalents.

### Sperm DNA fragmentation by terminal deoxynucleotidyl transferase-mediated fluorescein-dUTP nick end labeling (TUNEL) assay

Terminal deoxynucleotidyl transferase-mediated fluorescein-dUTP nick end labeling (TUNEL) using the Apo-Direct™ kit (Pharmingen, San Diego, CA) was used to measure the extent of DNA damage as described in earlier studies [26,27]. Briefly, 1–2 million spermatozoa were resuspended in 3.7% paraformaldehyde and re-suspended in 70% ice-cold ethanol. Positive and negative kit controls as well as internal controls (specimens from donors and patients with known DNA damage) were included for each run. The staining solution contained terminal deoxytransferase (TdT) enzyme, TdT reaction buffer, fluorescein isothiocynate tagged deoxyuridine triphosphate nucleotides (FITC-dUTP) and distilled water. All specimens were further washed in rinse buffer to remove the unbound reaction solution, re-suspended in 0.5 mL of propidium iodide/RNase solution, and incubated for 30 minutes in the dark at room temperature followed by flow cytometric analysis.

All fluorescence signals of labeled spermatozoa were analyzed by the flow cytometer FACScan (Becton Dickinson, San Jose, CA). A total of 10,000 spermatozoa were examined for each assay at a flow rate of <100 cells/sec. The excitation wavelength was 488 nm; green fluorescence (480–530 nm) was measured in the FL-1 channel and red fluorescence (580–630 nm) in the FL-2 channel using the flow cytometer software FlowJo Mac version 8.2.4 (FlowJo, LLC, Ashland, OR) [26,27].

Semen samples were divided into ROS+ and ROS- groups. All samples were frozen at −55°C until the time of analysis without addition of any cryoprotectant.

### Trypsin digestion

For proteomic analysis, seminal plasma was separated after cryopreservation. To prepare the samples for proteomic analysis, samples were thawed, and seminal plasma was separated from the sperm pellet by centrifugation at 3,000 g for 30 minutes to ensure complete removal of the cellular components. Seminal plasma samples were pooled and dissolved in 98% acetonitrile containing 0.1% trifluoroacetic acid followed by lyophilization at -80°C under vacuum for 2 days. The lyophilized sample was used to estimate the protein content. The samples were first precipitated in cold acetone, solubilized in 6 M urea, reduced with dithiothreitol and alkylated with iodoacetamide. The samples were subsequently diluted to give a urea concentration <2 M and then digested with trypsin. The tryptic digested products were subjected to a C18 clean up and then brought up in 50 μL of 1% acetic acid.

### Liquid chromatography- mass spectrometer analysis (LC-MS-MS)

The LC-MS/MS system used in this study was a Finnigan LTQ linear ion trap mass spectrometer. Ten μL volumes of the extract were injected on a self-packed high performance liquid chromatography column (Phenomenex Jupiter C18 reversed-phase capillary chromatography column). Peptides were eluted from the column by an acetonitrile/0.1% formic acid gradient at a flow rate of 0.25 μL/ min. They were introduced into the source of the mass spectrometer on-line. The microelectrospray ion source was operated at 2.5 kV. The digest was analyzed using the data dependent multitask capability of the instrument acquiring full scan mass spectra to determine peptide molecular weights and product ion spectra to determine amino acid sequence in successive instrument scans.

### Data analysis

The data was analyzed using all collision-induced dissociation spectra collected in the experiment to search the National Center for Biotechnology Information (NCBI) human reference sequence database with the search program MASCOT, a mass spectral search algorithm that uses mass spectrometry data to identify proteins from primary sequence databases. (Mascot version 2.7; Matrix Science, Boston, MA). These searches were used to identify the proteins present in the in-solution digestions. Following this, a second set of search was performed with SEQUEST which is bundled into Proteome (Discoverer 1.3; Thermo Finnigan), a tandem mass spectrometry data analysis program used for protein identification. Sequest identifies collections of tandem mass spectra to peptide sequences that have been generated from databases of protein sequences. The results from these SEQUEST searches were used to determine the spectral counts. Normalization of the spectral count was done using the total number of spectral counts for all proteins in the sample and the number of amino acids present in the protein. A 2-fold change in protein expression was considered significant since the precision of the proteomic analysis has an average error of 10-20%.

Functional bioinformatics analysis was done using publicly available (Gene Ontology (GO) annotations from GO Term Finder [28] and GO Term Mapper), UNIPROT [29], STRAP [30], BioGPS [31] and proprietary software packages such as Ingenuity Pathway Analysis (IPA from Ingenuity® Systems), Metacore™ (GeneGo Inc.) and STRING database [32] and Cytoscape [33] to identify the differentially affected processes, pathways, cellular distribution, and protein-protein interactions amongst proteins in the two study groups as well as for data integration.

### Pathway and network analysis

IPA and Metacore™ pathway databases were used to gain a deeper understanding into the pathways, networks and molecular and cellular functions affected in ROS+ and ROS- groups. The NCBI accession number and/or gene symbols were used as input identifiers for the analysis of proteomic dataset using IPA and IPA reported top networks, pathways, and biological functions significant to the protein dataset in terms of p-values and/or a score.

The significance of the association between the dataset and the canonical pathway was measured by comparing the number of specific proteins of interest that participate in a given pathway to the total number of occurrences of these genes in the dataset and the canonical pathway is explained only by chance. The 'ratio' associated with each pathway indicates the percentage of proteins in a pathway that were also found in our uploaded proteins list. The IPA network Score (used to rank networks according to the degree of relevance to the network eligible molecules in the user dataset) is based on the hypergeometric distribution and is calculated with the right-tailed Fisher's Exact Test.

Similarly, the Metacore™ reported statistically significant pathways, process or metabolic networks based on false discovery rate (threshold of P<0.05). The dataset was further subjected to identify protein functional network analysis using STRING software that identified both known and/or predicted interactions amongst the proteins based on experimental and curated databases.

## Results

### Semen parameters and oxidative stress parameters

The results for semen parameters, ROS, TAC and DNA damage is shown in Table 1. Sperm concentration (×10^6^/ mL) and percent motility in the controls was 69.49 ± 42.95 and 55.4% ± 14.0%, respectively, compared to 42.93 ± 52.84 and 49.1% ± 16.4% in the infertile men. Among the patients, 46.8% (15/32) had > 1 × 10^6^ round cells / mL compared to the donors 35% (7/ 20), and of these, 16% were positive for the Endtz test, a marker for granulocytes (the major contributor of ROS production). None of the donors was positive for the Endtz test. An overlap in morphology was seen in both donors (3.5% ± 1.6%) and infertile men (3.6% ± 3.2%).

**Table 1:** Semen parameters for donors, patients and ROS+ and ROS- samples

**Parameter**	**Donor (n = 20)**	**Patients**	**ROS-**	**ROS+**
		**(n = 32)**	**(n = 20)**	**(n = 32)**
*Sperm concentration*	69.49 ± 42.95	42.93 ± 52.84	59.36 ± 45.61	43.51 ± 35.46
(×10^6^/mL)				
*Motility* (%)	55.4 ± 14.0	49.1 ± 16.4	51.6 ± 14.2	51.0 ± 16.7
*Round cells* (×10^6^/mL)	2.92 ± 2.48	5.15 ± 2.37	2.40 ± 2.92	2.18 ± 2.10
*Endtz (Myeloperoxidase positive)* (×10^6^/mL)	0.00 ± 0.00	0.00 ± 0.15	0.00 ± 0.00	0.10 ± 0.17
*Morphology Kruger’s strict criteria* (%)	3.5 ± 1.6	3.6 ± 3.2	3.2 ± 2.5	3.8 ± 2.8
*ROS* (RLU/sec/10^6^ sperm)	2219.3 (0.0, 4438.5)	282.0 (0.0, 564.0)	4.0 (0.0, 9.0)	22.4 (33.0, 4439.0)
*Median* (Min., Max)				
*ROS* (RLU/sec/10^6^ sperm)	4.4 (0.0, 300.2)	104.0 (0.0, 1341.0)	0.0 (0.0, 0.0)	503.8 (101.6, 1407.9)
*Median* (25^th^, 75^th^ percentile)				
*TAC* (Micromole Trolox)	-	2065.0 ± 5343.0	1994.0 ± 507.4	2011.0 ± 556.2
*DNA* (%)	-	23.8 ± 10.9	18.0 ± 13.7	13.8 ± 9.4

*ROS* reactive oxygen species, *RLU* relative light unit.

Levels of ROS were elevated in many of the donors. However, the levels were significantly lower (RLU/s/×10^6^ sperm; Median (25^th^, 75^th^ percentile) [4.4 (0, 300.2)] in the donors compared with those in the infertile men [104(0, 1341.0)]. Among the primary infertility patients with <2 years of infertility, 80% were positive for ROS versus 56% of the patients with >2 years of primary infertility. Of those diagnosed with secondary infertility, a significantly larger number of men had higher levels (>20 RLU) of ROS. Teratozoospermia was present in 48% of men with primary infertility. Antioxidant and DNA damage was measured only in the infertile group. Antioxidant levels were low (<2000 micromoles of trolox) in 48% of the men with primary infertility compared with 57% in the men with secondary infertility. A higher incidence of DNA damage (>19%) was seen in 28% of the infertile men with secondary infertility (up to 49% DNA damage) compared with 14% of the men with primary infertility.

For proteomic analysis, all samples were categorized as either ROS- i.e. <20 RLU/s/10^6^sperm: median (Min, Max) of 4.0 (0.0, 9.0) or 25^th^, 75^th^ percentile of 0.0 (0.0, 0.0) and ROS+ with ROS levels significantly higher; median (Min, Max) of [22.4(33.0, 4439.0)] or median, 25^th^, 75^th^ percentile of 503.8 (101.6, 1407.9). The two groups were significantly different (P<0.01).

### Identification of the proteins by mass spectrometry

We utilized LC-MS/MS to identify the proteins in seminal plasma obtained from seminal ejaculates with high levels of (ROS+) compared to those with normal levels (ROS-). A total of 14 proteins were identified in these samples seven of these were identified in both the ROS- and ROS+ groups (Table 2). A comparison of the proteins identified in the ROS+ and ROS- groups is given in Table 3. Three proteins were uniquely expressed in the ROS- group: fibronectin I isoform 3 preprotein/ fibronectin 1 isoform b precursor, macrophage migration inhibitory factor-1 peptide and galectin 3 binding proteins. There were 4 preproteins uniquely expressed in the ROS+ group: cystatin S precursor, albumin preprotein, lactotransferrin precursor-1 peptide and prostate specific antigen isoform 4 preprotein were identified only in the ROS+ group.

**Table 2:** Proteins identified in LC-MS/MS from ROS- and ROS+ seminal plasma

**No.**	**Protein name**	**NCBI database index number**	**Calaculated MW (kDa)**	**pI**	**ROS-**		**ROS+**	
					**Peptides**	**Mascot**	**Peptides**	**Mascot**
					**(Coverage)**		**(Coverage)**	
1	*semenogelin II precursor*	4506885	65	9.0	9 (20%)	1968	7 (10%)	6998
2	*semenogelin I isoform b preproprotein*	38049014	45	9.2	6 (14%)	1597	3 (14%)	345
3	*fibronectin 1 isoform 3 preproprotein*	16933542	262	5.4	5 (3%)	964	-	-
4	*prostate specific antigen isoform 1 preproprotein*	4502173	29	7.6	6 (38%)	486	3 (15%)	255
5	*clusterin isoform 1*	42716297	58	6.2	2 (3%)	233	4 (12%)	203
6	*macrophage migration inhibitory factor*	4505185	12	7.7	2 (9%)	206	-	-
7	*prolactin-inducible protein precursor*	4505821	16	8.2	3 (48%)	123	5 (44%)	11771
8	*galectin 3 binding protein*	5031863	66	5.1	1 (3%)	104	-	-
9	*albumin preproprotein*	4502027	71	5.9	-	-	7 (17%)	1092
10	*cystatin S precursor*	4503109	16	4.9	-	-	2 (32%)	188
11	*prostate specific antigen isoform 4 preproprotein*	71834855	24	7.0	-	-	2 (10%)	163
12	*zinc alpha-2-glycoprotein 1*	4502337	34	5.7	1 (7%)	125	2 (12%)	143
13	*lactotransferrin precursor*	54607120	80	8.5	-	-	1 (3%)	140
14	*acid phosphatase, prostate short isoform precursor*	6382064	45	5.8	3 (8%)	220	3 (8%)	316

**Table 3:** Identification of proteins unique to ROS+ and ROS- groups and those common to both groups

**Protein name**	**Gene**	**NCBI accession no.**	**UniProt accession no.**	**NSC ratio**	**Log2 NSC ratio**
*fibronectin 1 isoform b precursor*	FN1	16933542	P02751	ROS- only	
*macrophage migration inhibitory factor-1 peptide*	MIF	4505185	P14174	ROS- only	
*galectin 3 binding protein*	LGALS3BP	5031863	Q08380	ROS- only	
*cystatin S precursor*	CST4	4503109	P01036	ROS+ only	
*albumin preprotein*	ALB	4502027	P02768	ROS+ only	
*lactotransferrin precursor-1 peptide*	LTF	54607120	P02788	ROS+ only	
*prostate specific antigen isoform 4 preprotein*	KLK3	71834855	P07288	ROS+ only	
*prolactin induced protein*	PIP	4505821	P12273	12.711	3.668
*semenogelin II precursor*	SEMG2	4506885	Q02383	1.338	0.420
*acid phosphatase, prostate short isoform precursor*	ACPP	6382064	P15309	1.063	0.088
*clusterin preprotein*	CLU	355594753	P10909	0.753	−0.409
*zinc alpha-2-glycoprotein 1*	AZGP1	4502337	P25311	0.354	−1.498
*prostate specific antigen isoform 1 preprotein*	KLK3	4502173	Q546G3	0.124	−3.012
*semenogelin I isoform a preprotein*	SEMG1	4506883	P04279	0.032	−4.966

*FN1* Fibronectin 1 isoform 3 preprotein, *MIF* macrophage migration inhibitory factor-1 peptide/factor protein, *LGALS3BP* Galectin-3 binding protein, *CST4* cystatin S-type 4, *ALB* Albumin, *LTF* Lactoferrin, *KLK3* Prostate specific antigen, *PIP* Prolactin-inducing protein, *SEMG2* Semenogelin-II, *ACPP* Prostatic acid phosphatase / prostatic specific acid phosphatase, *CLU* Clusterin, *AZGP1* Zinc-alpha-2-glycoprotein 1, *SEMG1* Semenogelin-I.

The seven proteins that were commonly expressed in both groups were: the prolactin induced protein, semenogelin II precursor, acid phosphatase, prostate short isoform precursor, clusterin preprotein, zinc alpha-2-glycoprotein, prostate specific antigen isoform 1 preprotein and semenogelin I isoform (a preprotein). The relative expression of these proteins was determined by analysis of the spectral counts. This label free method utilizes the number of spectra observed for each protein as a measure of relative abundance. This type of analysis is commonly used in proteomic experiments [26,27]. The normalized spectral count (NSC) ratios between the ROS+ and ROS- samples as well as their log2 values of the NSC ratios are given in Table 3.

Upregulation (overexpressed) or downregulation (underexpressed) of a protein was calculated from the Log2 NSC ratio. Seven proteins were found to be differentially expressed (Table 3). The upregulated proteins in the ROS+ group included the prolactin-induced protein, semenogelin II precursor and acid phosphatase and prostate short isoform precursor. The proteins down regulated in the ROS- group included the clusterin preprotein, Zinc alpha-2-glycoprotein 1, prostate specific antigen isoform I preprotein and semenogelin I isoform a preprotein.

### Gene ontology annotations of the proteins identified in ROS+ and ROS- samples

**Figure 1:**
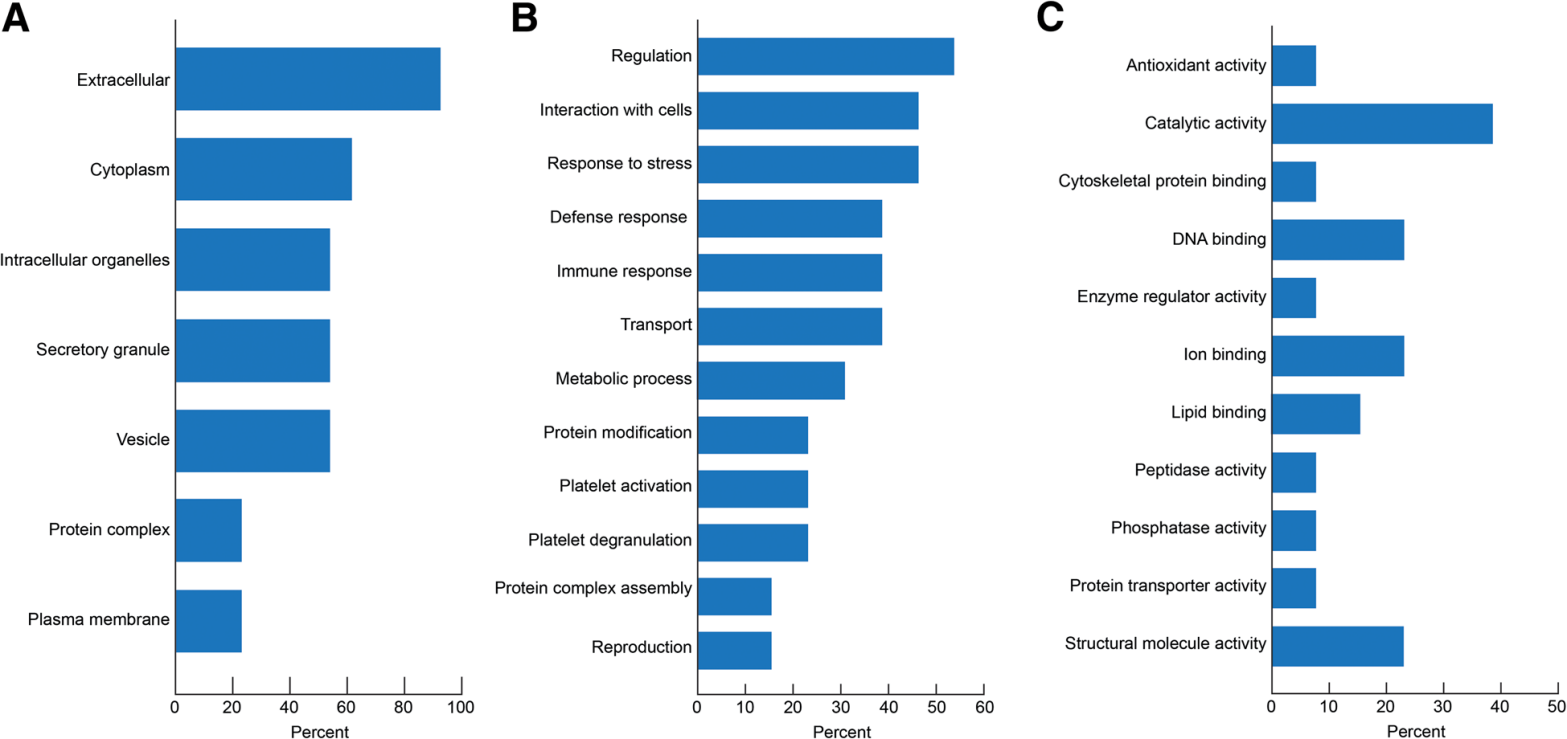
**Functional annotation from consolidated publicly available software tools. (A)**: Cellular distribution of seminal plasma proteins in ROS+ and ROS- group. Most proteins were localized in the extracellular region **(B)**: Biological processes distribution of seminal plasma proteins identified in ROS+ and ROS- group. Majority of the proteins were associated with protein regulation **(C)**: Molecular function distribution of seminal plasma proteins in ROS+ and ROS- group showing majority of the protein with catalytic activity.

Gene ontology analyses were performed to establish the link between cellular localization and biological processes for all the proteins expressed commonly or uniquely in the ROS+ and ROS- groups (Figure 1A-C). The cellular distribution analyses showed that most proteins were localized in the extracellular region while a smaller proportion of proteins originated from the plasma membrane region (Figure 1A). Similarly, the distribution of proteins in the biological processes showed a maximum involvement in stress and regulatory responses (Figure 1B). Furthermore, we were also able to show that most proteins were involved in catalytic activity (Figure 1C).

**Figure 2:**
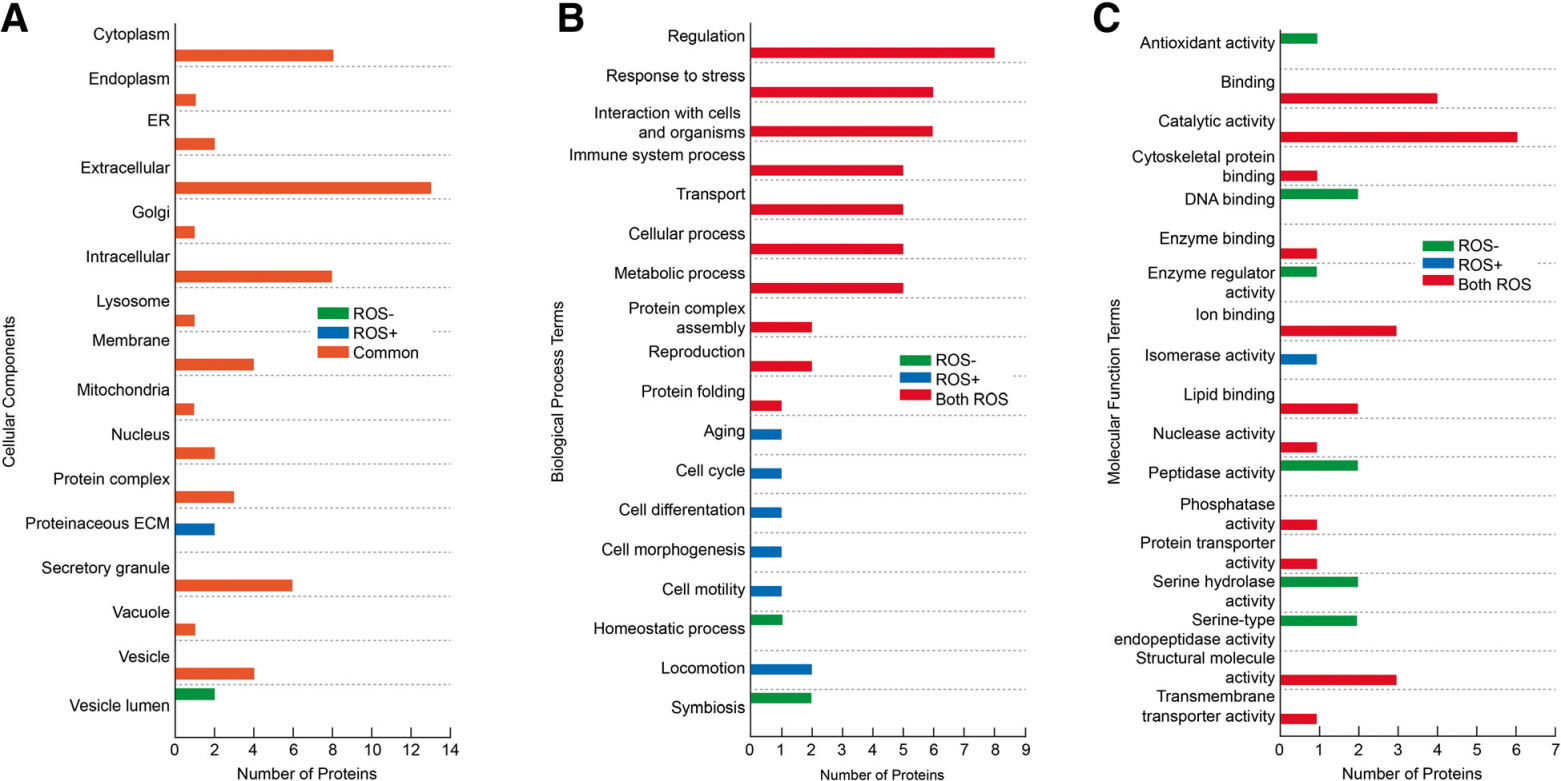
**Comparative analysis was done using GO annotations in 3 categories either unique to ROS+ and ROS- groups or common to both. (A)**: Among the cellular distribution of proteins the most commonly expressed in ROS+ and ROS- groups were extracellular in origin **(B)**: Biological processes of proteins commonly expressed in both ROS- and ROS+ group involved in major functions such as the regulation, response to stress, cellular and metabolic processes and reproduction **(C)**: Molecular functions of proteins that were common was involved in catalytic activity.

A comparative analysis was done using GO annotations to determine the distribution pattern of the proteins identified in the three categories that were either unique to ROS+ and ROS- groups or common to both. A significant overlap in the functional categories was observed for all the proteins in the ROS+, ROS- and commonly expressed groups. Among the unique proteins expressed in ROS+ group, two were involved in proteinaceous extracellular matrix (ECM) while the unique proteins in the ROS- group were restricted to the vesicular lumen region (Figure 2A).

The cellular distribution patterns revealed that most of the commonly expressed proteins were extracellular in origin (Figure 2A). Analysis of the biological processes revealed that the proteins commonly expressed in both ROS- and ROS+ group were involved in major functions such as the regulation, response to stress, cellular and metabolic processes and reproduction (Figure 2B). The unique proteins restricted to the ROS+ group revealed that they were involved in cell morphogenesis, cell motility, cell cycle and aging; these proteins were absent in ROS- group (Figure 2B).

Analysis of the molecular functions revealed that proteins uniquely expressed in the ROS- groups were involved in various enzymatic activities such as antioxidant activity, DNA binding, enzyme regulation, peptidase and serine hydrolase and serine endopeptidase activities; all of these functions were diminished in the ROS+ group (Figure 2C).

### Pathways and network analysis of proteins

Ingenuity Pathway Analysis (IPA) and Metacore™ were utilized to elucidate the biological pathways for proteins unique or common to the ROS+ and ROS- groups. Cancer, diseases of the reproductive system, immunological disease and inflammatory response were the top diseases or disorders. The molecular and cellular functions associated with the ROS- and ROS+ groups were cellular development, cellular growth and proliferation, cellular movement, cell-to-cell interaction and gene expression. Hair and skin development, development and function of the hematological system, immune cell trafficking, tissue development and tumor morphology were among the highly represented physiological and functional processes. The significant canonical pathways identified in our study are shown in Table 4.

**Table 4:** Significant canonical pathways associated with seminal plasma

**Ingenuity Canonical Pathways**	**Ratio**	**Proteins**
***Eumelanin Biosynthesis***	**5E-01**	**MIF**
***Role of Macrophages, Fibroblasts and Endothelial Cells in Rheumatoid Arthritis***	**1.35E-02**	**MIF, FN1**
*Intrinsic Prothrombin Activation Pathway*	8.33E-02	KLK3
*MSP-RON Signaling Pathway*	5.88E-02	KLK3
***MIF-mediated Glucocorticoid Regulation***	**4.76E-02**	**MIF**
***MIF Regulation of Innate Immunity***	**4E-02**	**MIF**
*Thyroid Cancer Signaling*	3.57E-02	KLK3
*Neurotrophin/TRK Signaling*	2.5E-02	KLK3
*Atherosclerosis Signaling*	1.89E-02	CLU
*Prostate Cancer Signaling*	1.79E-02	KLK3
*LXR/RXR Activation*	1.56E-02	CLU
*Human Embryonic Stem Cell Pluripotency*	1.56E-02	KLK3
*IL-12 Signaling and Production in Macrophages*	1.54E-02	CLU
***Hepatic Fibrosis / Hepatic Stellate Cell Activation***	**1.2E-02**	**FN1**
*Production of Nitric Oxide and Reactive Oxygen Species in Macrophages*	1.11E-02	CLU
***Acute Phase Response Signaling***	**1.06E-02**	**FN1**
*Clathrin-mediated Endocytosis Signaling*	1.06E-02	CLU
***ILK Signaling***	**1.05E-02**	**FN1**
***Actin Cytoskeleton Signaling***	**9.01E-03**	**FN1**

Pathways associated with proteins specific to ROS- group are in bold. *MSP-RON* Macrophage-stimulating protein- transmembrane receptor kinase, *MIF* Macrophage migration inhibitory factor, *TRK* Tyrosine kinase, *LXR/RXR* Liver X receptor / retinoid X receptor, *IL* Interleukin, *KLK3* Prostate specific antigen, *CLU* Clusterin, *FN1* Fibronectin 1 isoform 3 preprotein.

**Figure 3:**
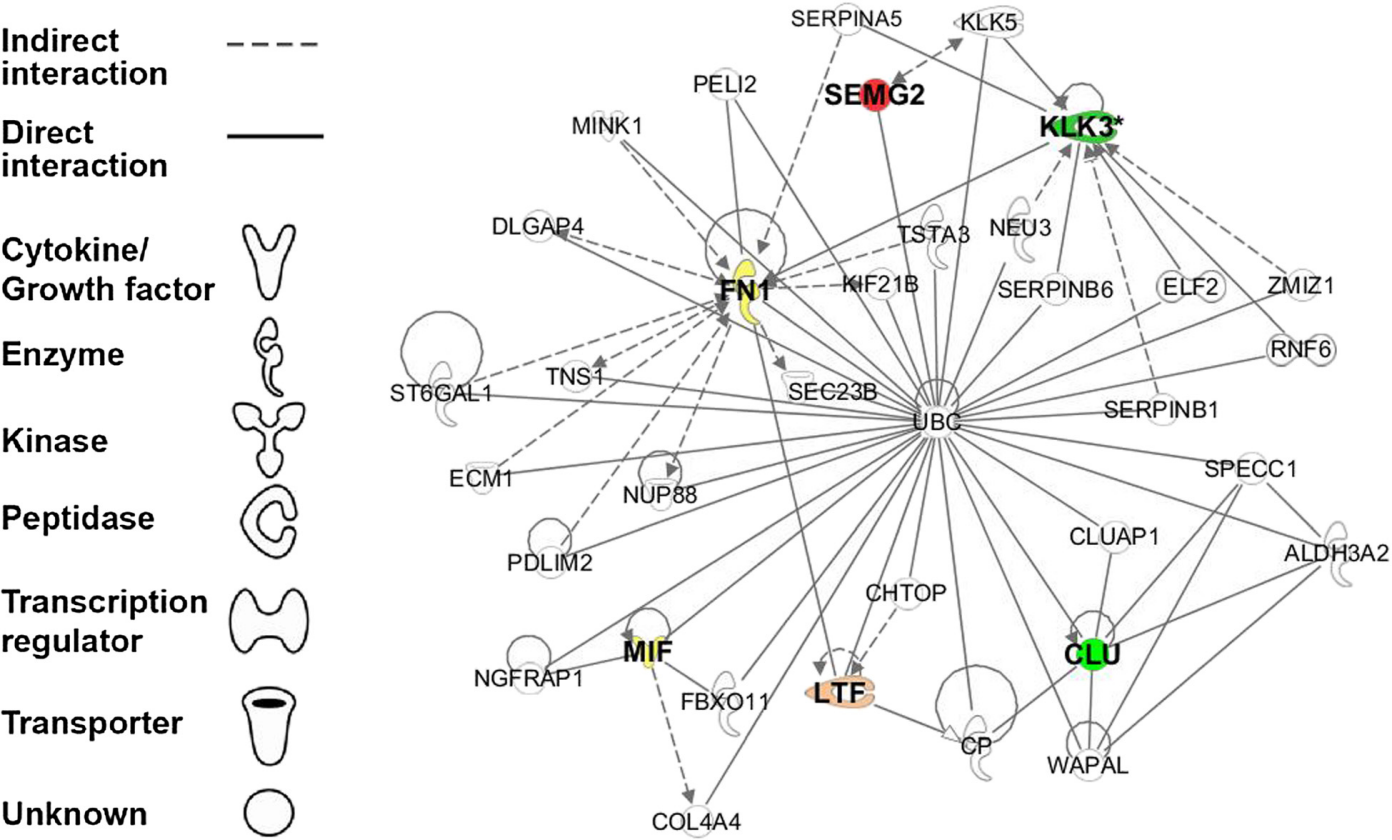
**IPA generated network for seminal plasma proteins showing 6 focus molecules highlighted in bold.** KLK3 = Prostate specific antigen; SEMG2 = Semenogelin-II; FN1 = Fibronectin 1 isoform 3 preprotein; LTF = Lactoferrin; MIF = macrophage migration inhibitory factor-1 peptide/factor protein and CLU = Clusterin.

**Figure 4:**
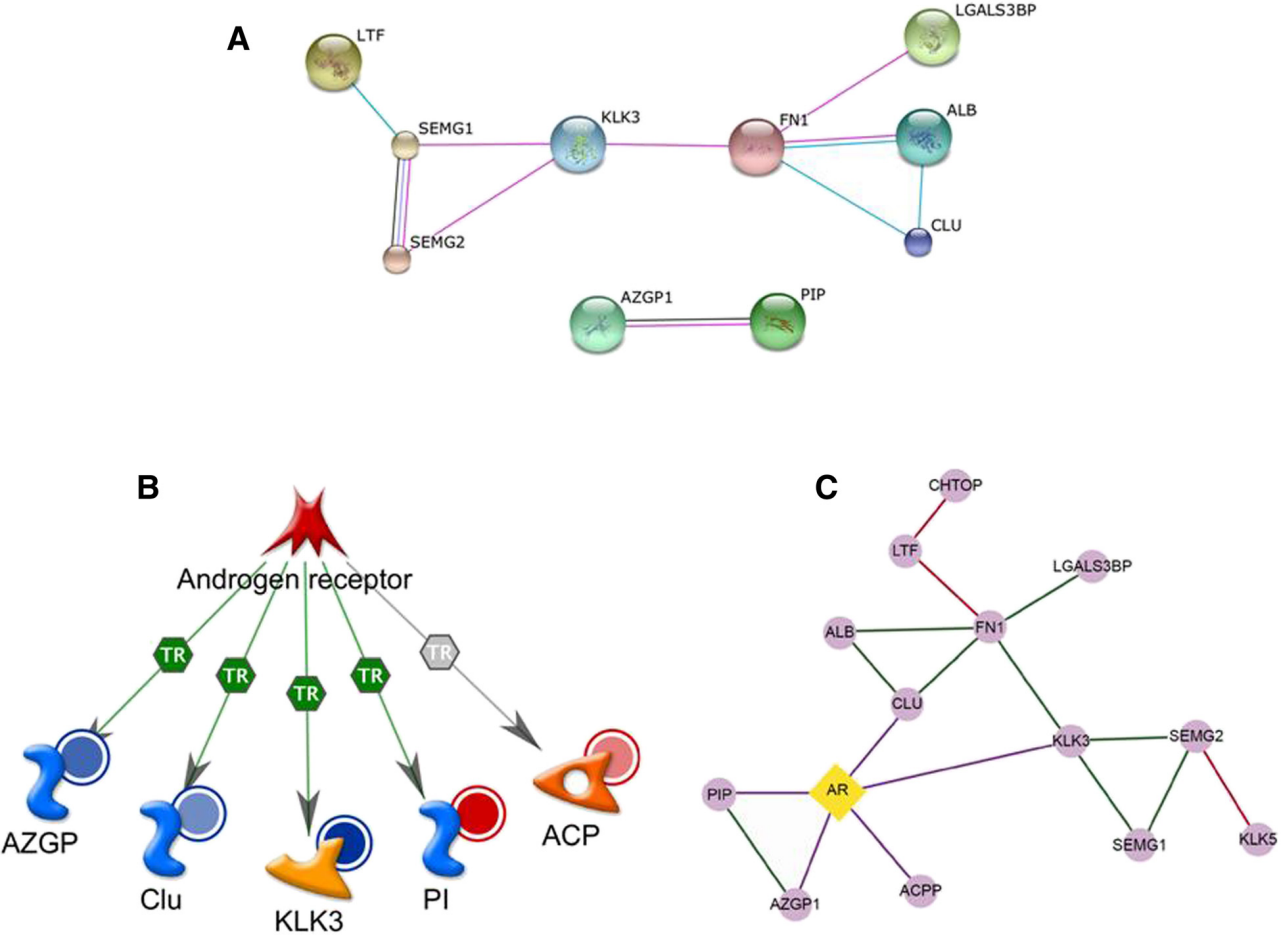
**Known or predicted protein-protein interaction network for seminal plasma proteins, A: 8 of 14 proteins interactions identified by STRING analyses were based on ‘experimental evidence’ and/or ‘curated pathway databases’.B**. Five proteins regulated by androgen receptor were identified through Metacore analysis; red shaded circles = upregulated proteins; blue circle = downregulated proteins, the shaded indicates the intensity of the regulation, **C**: Consolidated direct interaction network evidenced from 3 data sources (IPA, Metacore and STRING) showing that amongst 12 of 14 proteins, the central players were FN1, KLK3, and AR, all of which are connected to the majority of these proteins.

Fourteen proteins were analyzed with IPA software to identify the significant pathways and interaction networks. The top and the only network generated (with a network score of 14) was composed of 35 nodes (genes/proteins), only 6 of which were observed in our dataset of seminal plasma proteins (Figure 3). SEMG2 (Semenogelin-II) and CLU (Clusterin) proteins were significantly (2 fold) changed (up or down- regulated) in the ROS+ compared to the ROS- groups. FN1 (Fibronectin 1 isoform 3 preprotein) and MIF (macrophage migration inhibitory factor-1 peptide) were observed only in the ROS- group and LTF (Lactoferrin) was present only in ROS+ group. KLK3 (Prostate specific antigen) was observed in both the ROS+ and ROS- group. The common protein that connected 5 out of these 6 proteins was Ubiquitin C which is involved in conjugation and degradation of proteins impacting major processes and functions. The top functions of the network were cellular movement, cell-to-cell signaling and interaction, and infectious disease (Figure 4).

The Metacore analysis revealed the top pathways, process and regulatory networks for proteins that were common or unique to ROS+ and ROS- groups—these are shown in Table 5 and Figure 4A. Further, STRING database was used to identify any known or predicted direct interactions amongst these seminal proteins. The STRING database identified interactions between eight proteins (KLK3, SEMG1, SEMG2, FN1, LGALS3BP, ALB, PIP, and AZGP1) based on experimental evidence. However PIP and AZGP1 did not interact with the remaining six proteins. Five proteins known to be regulated by androgen receptor were identified through Metacore analysis (Figure 4B). The top GO processes identified for this network were male somatic sex determination, activation of prostrate induction by androgen receptor signaling pathway and negative regulation of integrin biosynthesis process (Figure 4C). The known direct interaction network amongst the proteins as evidenced by the three software packages, IPA, Metacore, and STRING, is shown in Figure 4C.

**Table 5:** Significant pathways, networks and processes identified from Metacore analysis of seminal plasma proteins

**Metacore pathways**	**Proteins (from our dataset) associated with the category**
*Protein folding and maturation*	KLK3
*Immune response*	CLU
*Androgen receptor signaling*	KLK3
*Extracellular matrix remodeling (ECM)*	KLK3
*ATP/ Ionisitol triphosphate metabolism*	ACPP
***Metacore process networks***	
*Reproduction*	KLK3, CLU
*Proteolysis ECM remodeling*	KLK3, CLU
*Proteolysis connective tissue degradation*	KLK3
*Inflammation*	CLU
*Signal transduction*	PIP, KLK3
*Reproduction feeding and neurohormone signaling*	PIP
*Immune response antigen presentation*	AZGP1
*Regulation of angiogenesis*	CLU
***Metabolic network***	
*Alpha-L-fucosyl (1–2)-D-galactose pathway*	KLK3, SEMG1, SEMG2
*Phosphatidylethanolamine pathway*	KLK3
*2-arachidonoyl-glycerol 3-phosphocholine pathway*	KLK3
*Lipid metabolism phospholipid metabolism*	ACPP
***Biological processes***	
*Insemination*	SEMG1, KLK3
*Liquefaction of seminal ejaculate*	KLK3
*Reproduction*	KLK3, CLU, SEMG1, SEMG2

*KLK3* Prostate specific antigen, *CLU* Clusterin, *ACPP* Prostatic acid phosphatase / prostatic specific acid phosphatase, *PIP* Prolactin-inducing protein, *AZGP1* Zinc-alpha-2-glycoprotein, *SEMG1* Semenogelin-I, *SEMG2* Semenogelin-II.

## Discussion

Although an increasing number of studies have identified more than 2545 unique proteins expressed in seminal plasma very few seminal plasma proteins have been unambiguously linked to infertility [34-36]. The role of ROS and its effect on the seminal plasma proteins has not been conclusively determined. A report by Wang et al., showed down regulation of certain proteins in the seminal plasma of asthenozoospermic patients as a result of oxidative stress, and attributed it to infection and environmental factors [12]. In the current study we utilized a combination of proteomic tools such as the LC-MS/MS and database searches to identify the proteins and the potential pathways/ networks that might be relevant to oxidative stress induced sperm dysfunction.

### MS identification of the proteins

We used two different search engines to decrease the amount of false positive protein identification and quantitative results. The utilization of multiple search engines is fairly common place and has been shown to improve the reproducibility and sensitivity of proteomic experiments [37]. Furthermore, the utilization of multiple search engines in these experiments was performed to verify the spectral count information and the proteins listed as quantitatively different were validated by both search engines. The searches were redone with newer versions of both Mascot and Sequest and the same proteins were identified in both searches eliminating any differences in the results. Biological replicates are important in these types of experiments. Therefore, the samples from 32 infertile men and 20 normal donors were characterized as either ROS- or ROS+ and pooled prior to digestion and LC-MS/MS analysis. The LC-MS/MS analysis was only carried out once and therefore no p-values were assigned. These experiments were analyzed with only one LC-MS analysis with the strategy that follow up validation experiments would be performed on additional biological replicates that would verify the LC-MS results.

Three of the important proteins - FN1 (Fibronectin 1 isoform 3 preprotein), macrophage migration inhibitory factor protein (MIF) and Galectin 3 binding protein (G3BP)-were solely expressed in the ROS- group and were distinctly absent in ROS+ group. Fibronectin is a ubiquitous multifunctional glycoprotein and a component of the seminal fluid and plays a key role in the formation of seminal gel following ejaculation [38]. It can bind to cellular components that are exposed when a spermatozoa is damaged and thus helps select abnormal spermatozoa [39]. While our studies reported the presence of fibronectin only in the ROS- groups, Davalieva et al. reported increased expression of fibronectin in azoospermic patients when compared with normozoospermic men [40].

MIF is a secretory protein of the epididymis that has a catalytic property. It maintains the thiol protein oxidoreductase status of sperm, which in turn influences their motility [41]. G3BP belongs to a family of galectin β-galactoside-binding proteins and is involved in immunomodulation, cell-cell and cell-matrix adhesion, and pathogen-host interactions [42]. Compared to the studies of Davalieva et al., G3BP was found to be upregulated in oligozoospermic patients [38]. Although it is a substrate for prostate specific antigen, reports on the functioning of G3BP in male infertility are lacking [43]. The proteins unique to the ROS+ group included the cystatin S precursor, albumin preprotein, lactotransferrin precursor-1 peptide and prostate specific antigen isoform 4 preprotein. Interestingly, most of the proteins identified as being unique to the ROS+ group represent proteins that are present in their precursor form, and are most likely indicative of post-translational problems [44].

Lactotransferrin is an important transport protein that has antimicrobial properties and forms sperm coating antigen whereas albumin functions as a reservoir for cholesterol. Albumin must be removed from the sperm membrane during capacitation [4,45]. Cystatins are known to control the activity of cysteine proteases and thus, they exist in several isoforms [4].

Amongst the various differentially regulated proteins identified, the two isoforms of semenogelin showed a contrasting expression, i.e. semenogelin II precursor was found to be similar in expression while the semenogelin I isoform--a preprotein-was found to be down regulated in the ROS+ group compared with the ROS- group. This result differs from that of another study that found no significant differences between the two isoforms of semenogelin I and II in asthenozoospermic groups compared to healthy donors under oxidative stress conditions [12]. However, the overexpression of both semenogelin I and II was reported in post-varicocelectomy patients largely due to oxidative stress [46].

Another striking finding of our study was the high abundance of prolactin induced protein (PIP) in the ROS+ group. Seminal plasma is known to have immunomodulating properties [47-49]. While no association was reported between the levels of total immunoreactive PIP in the seminal plasma and the fertility rate [50], contradictory results were seen in the seminal plasma proteome from azoospermic men [51] and in the sperm proteome from globozoospermic and asthenozoospermic men [44,52]. Similarly, PIP expression was increased in the azoospermic group compared to the oligozoospermic group and thus, PIP could serve as a potential biomarker in these cases [40]. Increased presence of PIP in human spermatozoa has been correlated with poor sperm quality [53]. Prostate specific antigen preprotein was restricted to the ROS+ group; the isoform 1 preprotein was downregulated in the ROS+ group compared to ROS- group.

### GO annotation analysis of the MS identified proteins

All the proteins identified in the study were subjected to GO analysis, which revealed a large overlap of proteins in the cellular localizations, and biological and molecular functions of the proteins. This phenomenon of protein overlap in GO analysis of seminal plasma has also been identified by various other researchers, indicating that proteins in the seminal plasma are involved in multiple functions [5,12,54,55]. The GO annotation analysis showed a greater distribution of proteins with extracellular and cytoplasmic origin. Furthermore, intracellular organelles, secretory granules and vesicles also showed a similar distribution of proteins in both the ROS+ and ROS- group (Figure 1A). In the biological processes, regulation followed by stress response and interaction with the cells was observed (Figure 1B), suggesting that seminal plasma proteins regulate spermatogenesis and spermatozoa as they transit through the epididymis [56]. Among the molecular processes, a major distribution was seen in the catalytic activity, which indicates that the major proteins are involved in proteolytic cleavage (Figure 1C).

Cysteine proteases belong to a group of proteases that are involved in various regulatory mechanisms such as intracellular catabolism of peptides and proteins [57] processing of proenzymes and prohormones [58-60]. Cysteine proteases play a modulatory role in the proliferation and destruction of tissues by malignant cells during tumor invasion and metastasis [60] and by microorganisms during infection [61]. Cystatins act as inhibitors and protect the host tissues against destructive proteolysis [62]. Molecular functions also reveal a great deal of binding activity. Glycosaminoglycans such as the heparin binding proteins (HBPs) of the seminal fluid have been reported to attach to the sperm surface and enhance capacitation. Several heparin binding proteins in the seminal plasma have been characterized and alterations in these proteins have been shown to result in male infertility [4]. In our study lactoferrin, isoforms of semenogelin and prostate specific antigen precursor were similar to the many proteins characterized for heparin binding.

### Comparative GO annotation analysis of the proteins identified in ROS+ and ROS - groups

The cellular distribution pattern revealed that besides the common proteins, which were abundant in the extracellular region, the unique proteins of the ROS+ group were involved in extracellular matrix (ECM) formation while those of the ROS- group were in vesicle lumen (Figure 2A). This finding suggests that the unique proteins of the ROS- group assist in the process of endocytosis, which is diminished in the ROS+ group and making it more susceptible to infection and other inflammatory responses. Prostate specific antigen (KLK3) is known to be involved in ECM remodeling. In our study, two isoforms of KLK3 were observed; one was restricted to the ROS+ group whereas the other form was downregulated in the ROS+ group. However, both isoforms were present in preprotein forms, which is most likely caused by an improper post-translational process leading to an improper formation of the ECM complex. The comparative distribution of proteins in biological processes involved with ageing, cell cycle, cell differentiation and morphogenesis, motility and locomotion were seen only in the ROS+ group, suggesting that these functions may be involved in sperm interaction and therefore may play a role in sperm apoptosis, necrosis and cell death (Figure 2B).

The distribution pattern of molecular functions revealed that the common proteins had a high degree of involvement in catalytic activity. MIF is known for its catalytic property, which maintains the thiol redox status in spermatozoa. During epididymal transit, the histones in a mature spermatozoon are replaced with thiol groups to aid in the tight packaging of chromatin material. Its absence in the ROS+ groups indicates improper packaging of chromatin and thus DNA damage.

Our study revealed an important prostasome in the ROS- group, the Galectin-3 binding protein (LGALS3BP) which could represent an important candidate biomarker of reproduction and prostate cancer. Interestingly, while LGALS3BP was only restricted to the ROS- group, the semenogelin I and PIP proteins were differentially expressed. This is indicative of an improper binding regulatory activity [63].

### Pathway and network analysis of the identified proteins in ROS+ and ROS- groups

The IPA figure was generated to identify the indirect interactions or the shortest path that connects the proteins of our interest. The Metacore network was generated to identify the co-regulators or shared transcriptional regulators of the genes encoded by the proteins of our interest. Since there were a significant number of proteins regulated by androgen receptor (AR), the STRING network was generated to find out if any and which of these proteins directly interact with each other.

We chose to conduct multiple network analyses to accomplish different outcomes individualized for the respective questions we wanted to address. If we had merged the three networks as is into one, we would have compromised the clarity of figures; and missed on the elaboration of certain aspects of the network such as other interacting proteins (in the IPA figure) that were not observed in our samples, but are known to interact with our list of proteins and affect certain processes/functions. IPA pathway analysis of the commonly and differentially expressed proteins revealed that extracellular matrix proteins such as fibronectin were involved in the ILK (Integrin linked kinase) pathway. The binding of the fibronectin peptide could augment integrin signaling [64,65] during fertilization. Furthermore, Ingenuity Canonical Pathway (ICP) analysis showed that FN1 was involved in ILK signaling in the ROS- group and that this process may have been absent in the ROS+ group due to the absence of fibronectin. The shortest path connecting the majority of the proteins was through ubiquitin C, to which 5 out of the 6 proteins were known to bind (Figure 3).

Metacore™ analysis shows that KLK3 is a key protein that is involved in a majority of pathways, processes and networks. We have demonstrated important networks such as reproduction, proteolysis in remodeling of extracellular matrix, proteolysis in degradation of connective tissue and signal transduction. The metabolic networks that include KLK3 are alpha-fucosyl (1–2)-D galactose pathway, phosphatidylethanolamine pathway, 2-arachidonoyl-glycerol 3 phosphocholine pathway. The biological processes include insemination and seminal clot liquefaction. KLK3 or prostate specific antigen is secreted from the prostate secretion, and may play a role as a biomarker in prostate cancer [11].

Isoform 4 preprotein was specific to the ROS+ group only while the isoform 1 preprotein was downregulated in this group compared to ROS- group. It is likely that oxidative stress biomarkers can also be conceivably useful in identifying the underlying cause(s) of prostate cancer as it has been shown to exist in five different forms and 1 truncated form and hence, the lack of its specificity as a unique biomarker [11,66,67].

We also studied the interactions between the identified proteins using STRING software. We found that KLK3 regulated SEMG1 and SEMG2 as well as FN1. KLK3 was restricted only to the ROS+ group, suggesting improper cleavage of semenogelin and fibronectin and subsequent problems with liquefaction and hyperviscosity in this group [68,69]. Contrary to reports of Kumar et al., [70] who examined the interaction between human serum albumin and PIP, our STRING analyses did not show any interaction between albumin preprotein and PIP.

Our study through Metacore™ analysis also identified five genes that are transcriptionally regulated by the androgen receptor: AZGP1, Clusterin, KLK3, PIP and ACPP. Further analysis showed that these signaling pathways were not only involved in the activation of prostate induction but also in male somatic sex determination and negative regulation of the integrin biosynthetic process. Our consolidated direct interaction network (Figure 4C) amongst 12 of 14 proteins shows the central players as FN1, KLK3, and AR, all of which are connected to the majority of these proteins. The integrated seminal plasma protein direct interactome network of seminal plasma proteins in ROS+ and/or ROS- samples obtained from three data sources, (IPA, Metacore, and STRING) is shown in Figure 4C. FN1 interacts with 4 proteins - LGALS3BP, ALB, KLK3, and CLU interactions that are experimentally validated as per the STRING database and LTF and KLK3 interactions from IPA are evidenced from the literature curation efforts. The interactions shown by Metacore are the proteins (ACPP, AZGP1, CLU, KLK3, and PIP) encoded by genes that are regulated by androgen receptor. KLK3 also interacts with SEMG I, SEMG II, and FN1.

There were some limitations to our study. One of the possible drawbacks of our study was that we did not completely eliminate the presence of round cells (possible extrinsic source of ROS production in these samples). Round cells especially the granulocytes are significant contributors of ROS in the seminal ejaculate. We did not look into other information such as patient’s age/ethnicity, pharmacological treatments and medical status as our primary focus was to examine the effect of high and physiological levels of ROS on protein distribution irrespective of other criteria. Another limitation was the small number of proteins identified in the ROS+ and ROS- groups. This may have occurred due to masking of the low abundant proteins by the high abundant proteins--proteomic methods based on LC-MS/MS do not identify all biologically relevant components and they exclude analysis of intact proteins. Revalidation of the results by quantitative western immunoblot analysis of sperm extracts with specific antibodies is important. This was a pilot study for proof of concept. We plan to perform the immunoblot in our ongoing series of investigations.

## Conclusions

In conclusion, it is evident that proteomic analysis of seminal plasma has potential clinical implications. We have established the presence of several proteins including fibronectin and prostate specific antigen and PIP in seminal plasma with both low and high levels of ROS. The proteomic approach utilized in our study employing various bioinformatic tools revealed the cellular pathways that are involved in oxidative stress that could serve as biomarkers in identifying non-invasive diagnostic tools for various reproductive disorders. The relatively high abundance of PIP proteins needs to be further validated through Western Blot analysis as a possible biomarker for male infertility. It is important to understand the proteins involved in oxidative stress and inflammation because this will help identify any drugs and antioxidant therapies that could help treat infertile men with oxidative stress-related infertility.

## Abbreviations

ABTS: 2,2’-Azino-di-[3-ethylbenzthiazoline sulphonate]; ACPP: Prostatic acid phosphatase / prostatic specific acid phosphatase; ALB: Albumin; AR: Androgen receptor; ATP: Adenosine triphosphate; AZGP1: Zinc-alpha-2-glycoprotein; CHTOP: Chromatin target of protein PRMT1 (protein arginine N-methyltransferase 1); CLU: Clusterin; DNA: Deoxyribonucleic acid; dUTP: Deoxyuridine triphosphate nucleotide; ECM: Extracellular matrix; FITC: Fluorescein isothiocynate; FN1: Fibronectin 1 isoform 3 preprotein; G3BP: Galectin 3 binding protein; GO: Gene Ontology; HBPs: Heparin binding proteins; ICP: Ingenuity Canonical Pathway; IL: Interleukin; ILK: Integrin linked kinase; IPA: Ingenuity Pathway Analysis; IRB: Institutional Review Board; KLK3: Prostate specific antigen; LC-MS: Liquid chromatography-mass spectrometry; LC-MS/MS: Liquid chromatography-tandem mass spectrometry; LGALS3BP: Galectin-3 binding protein; LTF: Lactoferrin; LTQ: Linear trap quadrupole; LXR/RXR: Liver X receptor/retinoid X receptor; MALDI-TOF: Matrix assisted laser desorption ionization-time of flight; MASCOT: A tandem mass spectrometry data analysis program from Matrix Science that is used for protein identification; MIF: Macrophage migration inhibitory factor-1 peptide/factor protein; MS: Mass spectrometry; MSP-RON: Macrophage-stimulating protein-transmembrane receptor kinase; NCBI: National Center for Biotechnology Information; NSC: Normalized spectral count; PIP: Prolactin-inducing protein; RLU: Relative light units; ROS: Reactive oxygen species; SEMG1: Semenogelin-I; SEMG2: Semenogelin-II; SEQUEST: A tandem mass spectrometry data analysis program used for protein identification; STRING: Search Tool for the Retrieval of Interacting Genes/Proteins; STRAP: Serine-threonine kinase receptor-associated protein; BioGPS: A gene annotation portal; TAC: Total antioxidant capacity; TdT: Terminal deoxytransferase; TR: Transcription regulation; TUNEL: Terminal deoxynucleotidyl transferase-mediated fluorescein-dUTP nick end labeling; UNIPROT: Universal protein resource (a central repository of protein data combining Swiss-Prot, TrEMBL and PIR-PSD databases); WHO: World Health Organization.

## Competing interests

The authors declare that they have no competing interests.

## Authors’ contributions

RK: participated in the study conception/design, review of the data and writing of the manuscript and final approval; SD: acquisition and preparation of samples for analysis; BW: acquisition of samples for analysis; interpretation of the results; SY: acquisition of the samples, interpretation of the data, discussion of results; BG: contributed to bioinformatic analysis, data interpretation and participated in the paper redaction; ES: drafting of the article; GM participated in the review of the data and writing of the manuscript; AA: contributed to the study design, and review of the data. All authors read and approved the final manuscript.
